# Impact of an online training tool on individual and organizational resilience and mindfulness among radiological personnel in Norway

**DOI:** 10.1186/s13104-023-06659-7

**Published:** 2023-12-19

**Authors:** Ann Mari Gransjøen

**Affiliations:** 1 Department of Health Sciences in Gjøvik, Norwegian University of Science and Technology in Gjøvik (NTNU), Teknologiveien 22, Gjøvik, 2815 Norway; 2 SHARE-Centre for Resilience in Healthcare, Faculty of Health Sciences, University of Stavanger, Kjell Arholmsgate 41, Stavanger, 4036 Norway

**Keywords:** Resilience, Mindfulness, Radiological personnel, Radiology departments

## Abstract

**Background:**

Heavy workloads and increasing demands for productivity have contributed to rising rates of stress and burnout among radiological staff. Different forms of mindfulness and resilience-training might assist with stress management and protect these employees against burnout.

**Aim:**

The objective of this study is to evaluate the impact of an online training tool on individual and organizational resilience, mindfulness and quality of care.

**Methods:**

An online questionnaire was used, consisting of the Connor-Davidson Resilience Scale, the Mindful Attention Awareness Scale, the Benchmark Resilience Tool, and questions pertaining to quality, safety, and burnout (baseline = 68 participants between July 2022 - October 2022, follow-up = 13 participants between November 2022 - February 2023). Descriptive statistics and a paired-sampled t-test were used for statistical analysis.

**Results and conclusions:**

Few participants reported completing any of the exercises. The baseline group had significantly higher mean resilience (*p* = 0.018) and mindfulness scores (*p* = < 0.001), mean decrease in scores was 7.46 for resilience and 1.7 for mindfulness. In conclusion, both individual and organizational resilience are perceived as low among radiological personnel in Norway. However, it does not seem to affect quality and safety.

## Introduction

The increased use of diagnostic imaging [1–5] have led to an increased workload for radiological personnel. This can lead to increased rates of stress and burnout [6–9]. Two strategies that have been used to protect radiology staff against burnout has been mindfulness and resilience-training [8–11].

Mindfulness-training can consists of formal meditation and storytelling exercises or a more informal attentiveness to day-to-day tasks [9]. Resilience can be promoted through acknowledgement, exercises designed to improve resilience [10–12], professional autonomy, and recognition for accomplishments [10, 11]. Organizational changes can also promote resilience [10, 11].

Organizational resilience can facilitate employee resilience [13], the capability to cope with stress and stressors, learning at the individual and team level, and team efficacy [13, 14]. It is suggested that individual resilience contributes to organizational resilience due to its positive impact on transformation (resilient individuals help drive positive change in the organization) [13], positive impact on team efficiency [13], and there are some indications that supporting the individuals wellbeing is crucial for maintaining organizational resilience [15].

The aim of this study was to evaluate the impact of an online training tool on individual and organizational resilience, mindfulness and quality of care. The purpose of the training tool was to increase individual resilience and mindfulness among radiological personnel, offering a buffer against stress.

## Materials and methods

In collaboration with the Norwegian Society of Radiographers, and the Norwegian Radiological Association, data for the baseline measurement collected between July and October 2022. The follow-up measurement was collected from November 2022 to February 2023. These associations posted the link to the digital questionnaire on social media and in their newsletter to all members. These posts and newsletters were both the recruitment and reminder to use the online tools.

The online training tool consisted of three resilience exercises (three good things, upside of stress and self-compassion), three mindfulness exercises (breath as anchor, body-scan, and walking meditation), and offered background information (definitions of mindfulness, stress and stress management, what feelings are and how to identify them). All of these materials were available online on the WordPress platform in the form of short videos, audio-guides, and documents.

The questionnaire consisted of six parts. Part 1 was designed by the researcher, to collect data for background variables, such as the respondent’s profession, size of the departments by number of labs, and whether they work in the public or private sector. This information was used to assess whether any of the professions were more resilient than the others, and whether organizational resilience could differ between the different sectors.

Part 2 is the Norwegian Connor-Davidson Resilience Scale (CD-RISC-10). This scale is used to assess an individual’s ability to respond and adapt to life adversity and major life stressors [16].

Part 3 is the five question Mindfulness Attention Awareness Scale (MAAS), translated from English into Norwegian by Smith et al. This scale measures the extent to which an individual can attend to, and remain aware of, experiences in the present moment [17].

Part 4 is the short version of the Benchmark Resilience Tool (BRT 13), used to measure organizational resilience. This tool specifically assesses behavioral traits and perceptions linked to the organization’s ability to plan for, respond to, and recover from emergencies and crises [18]. The researcher, following the steps described by the Norwegian Directory of Health, translated the BRT 13 from English to Norwegian.

Parts 5 and 6 of the questionnaire were written for specific groups. Part 5 was intended for respondents with personnel management roles, and was only made available for those who reported being in such roles. This part of the questionnaire was inspired by the questionnaire developed by Parikh et al. (2020) to evaluate a manager’s effectiveness in detecting burnout among employees [19], and was translated from English to Norwegian.

Part 6 was intended for radiographers and radiotherapists, so it was made available only to respondents listing these as their profession. The researcher designed the questions to evaluate the aspects of quality and safety in radiology which may be affected by stress and mindfulness. These aspects of quality of care are self-reported by the respondents, and thereby their subjective experience, not objective quality indicators, key figures or patient reports.

Data analysis consisted of a paired t-test to compare means in the before and after groups. Due to lack of response to the follow-up questionnaire from radiographers and those working in the private sector, the researcher decided only to compare radiologists and trainees working in the public sector, making the groups compared as similar as possible. Thirty-two of the 68 questionnaires from the baseline were used. All analyses were performed using IBM SPSS version 29.0.

## Results

According to the analytics page provided by YouTube, the resilience exercises were accessed between two (Upside of stress) and 13 times (Three good things). The mindfulness exercises were available only through the WordPress-platform, which does not offer such analytics, so it was impossible to know how many times they were accessed.

There were 68 respondents to the baseline questionnaire, where 88% worked in the public sector, 7% worked in the private sector, and 4% worked in both. The majority worked either as a radiologist (46%) or radiographer (35%). Most respondents worked in departments with > 20 labs (31%). Eleven participants (16%) had a leadership role.

There were 13 respondents to the follow up-questionnaire, all of whom worked in the public sector. Of these 69% were radiologists and 31% were registrars, 46% worked in relatively large departments (16–20 labs), and 15% had a leadership role. Two participants (15%) reported having used any of the exercises provided in the online training tool.

A paired-samples t-test was conducted to evaluate the impact of the online training tool on the scores for individual resilience, mindfulness, organizational resilience and quality and safety. There was a significant difference between the groups for the scores for individual resilience and mindfulness. The baseline group had a higher mean resilience (Mean = 29.77, SD = 6.04) and mindfulness (Mean = 3.94, SD = 0.91) than the follow-up group (Mean = 22.31 and 2.20, SD = 6.09 and 0.46 respectively), *p* = 0.018 and < 0.001 respectively.

The mean decrease in resilience scores was 7.46 with a 95% confidence interval ranging from 1.51 to 13.41. The mean decrease in mindfulness scores was 1.7, with a 95% confidence interval ranging from 1.05 to 2.42. The eta squared statistics (0.22) indicates a large effect size.

## Discussion

There are some contradictory findings between this study and previous ones. One of the reasons for this can be that there were no strict program to be followed in the current study, but rather an online tool that was provided that participants could use as they chose. Similar studies has provided a structured training program for increasing resilience [12].

The choice to make an online training tool rather than using a strict program was based on several factors. Firstly, it was important to have a form of education regarding the tools that could be used. Not only in previous resilience studies [12], but also implementation research, education has often been seen as a factor for success [20–24]. Another factor was that the training should not be seen as an extra task that they are demanded to perform on top of existing work, but rather something to be done voluntarily when they had time [25]. In-person meetings are usually evaluated positively [26]; however, it is often difficult to achieve high attendance in such meetings due to the nature of working in diagnostic imaging.

The choice to not have a structured program may have contributed to the lack of use. It is interesting to note that even though it was not widely used, some participants still commented that learning tools for stress management were important, since diagnostic imaging is a somewhat stressful field indicating that such a tool might be useful.

The large, negative effect seen on both types of resilience and mindfulness is likely not caused by the online training tool, but rather by outside factors such as the timing of the project, which coincided with the last months of the COVID-19 pandemic, and the roll-out of a new digital system in a large part of Norway.

These factors are likely to have contributed to the lack of responses to the follow-up questionnaire, and the reduction in perceived resilience and mindfulness. Some participants in the follow-up questionnaire even claimed that the roll-out of the new digital system increased their stress, lowered their sense of personal accomplishment, and made them feel that they were just “going through the motions”, all of which may have contributed to the decline in mindfulness and resilience-scores.

It is also interesting to note that there are no significant changes in organizational resilience, which contradicts previous studies that show a correlation between these factors [13, 14, 27, 28]. Based on the correlation seen between individual and organizational resilience in these studies, some change in organizational resilience as perceived individual resilience were lower would have been expected.

When it comes to quality and safety, there were no significant changes, which was unexpected based on the results of previous studies [9, 29, 30]. Several studies indicate a correlation between mindfulness and quality of care [9, 29, 30], which does not seem to be true in the present study. If there was a significant correlation between the factors in this study, there quality and safety score should have a significant change with the considerably lower mindfulness score in the follow-up group. These findings however are consistent with the results from the study on the baseline group (Gransjøen, under review) where there were significant correlations between mindfulness and resilience, and between individual and organizational resilience.

In conclusion, the results of this study suggest that both individual and organizational resilience are perceived as low among radiological personnel in Norway. However, even if there was a reduction in resilience and mindfulness, it had no effect on quality and safety, indicating less of a correlation between these factors than in previous studies.

It could be beneficial to be more structured in offering radiological personnel stress management tools such as resilience exercises, and mindfulness exercises. As a result, they would have a more efficient way of learning how to use these tools, and the number of personnel implementing them in their work-lives would be more likely to increase.

### Limitations

There are several limitations to this study. The most serious of these are the small sample size and lack of a structured training program. Because of the small sample size the results are less valid and reliable than they would have been with a larger sample. Small sample sizes also limit the transfer value to the clinical setting, since a small sample is less likely to reflect the actual population.

The unstructured nature of the training program may have contributed to its lack of use. Since so few participants actually practiced any of the exercises, other factors such as the pandemic and changes in the workplace are likely to have had a larger effect on their resilience and mindfulness than the online training tool.

Another significant limitation is that it is not certain that those contributing to the baseline are those contributing to the follow-up. This is attributable to the anonymity of the study, where participants are assigned a random number by the website hosting the digital questionnaire, without any systems for connecting these numbers. For this reason, other types of t-tests tan the paired t-test might have been more appropriate for this study. However; the likelihood of participants being the same was high enough for a paired-samples t-test to be selected as the best fitting analysis.

Lastly, there is always the chance that those who are most frustrated with their jobs and the systems in place at their work answer the questionnaire, possibly skewing the results in a negative direction. If workers who are highly frustrated by, for example, the new digital systems are those who completed the questionnaire, this might explain the lower scores in all accounts in the follow-up group.

## Data Availability

The datasets used and/or analyzed during the current study are available from the corresponding author on reasonable request.

## Abbreviations

BRT: Benchmark Resilience Tool
CDRS: Connor-Davidson Resilience Scale
MAAS: Mindfulness Attention Awareness Scale
NSD: Norwegian Social Science Data Services

## References

[1] NHS England (2014). Diagnostic imaging dataset statistical release.

[2] NHS England (2020). Diagnostic imaging dataset annual statistical release.

[3] Smith-Bindman R, Kwan ML, Marlow EC, Theis MK, Bolch W, Cheng SY (2019). Trends in use of medical imaging in US health care systems and in Ontario, Canada, 2000–2016. JAMA.

[4] Winder M, Owczarek AJ, Chudek J, Pilch-Kowalczyk J, Baron J (2021). Are we overdoing it? Changes in diagnostic imaging workload during the years 2010–2020 including the impact of the SARS-CoV-2 pandemic.

[5] Hofmann BM, Gransjøen AM (2022). Geographical variations in the use of outpatient diagnostic imaging in Norway 2019. Acta Radiol Open.

[6] Ganeshan D, Wei W, Yang W (2019). Burnout in chairs of academic radiology departments in the United States. Acad Radiol.

[7] Giess CS, Ip IK, Gupte A, Dudley JC, Healey MJ, Boland GW (2022). Self-reported burnout: comparison of radiologists to nonradiologist peers at a large academic medical center. Acad Radiol.

[8] Kalantarova S, Mickinac N, Santhosh S, Malik S, Surovitsky M, Madsen L (2021). Preventing physician burnout in breast imaging: scope of the Problem and Keys to Success. Curr Probl Diagn Radiol.

[9] Spieler B, Baum N (2022). Burnout: a mindful framework for the radiologist. Curr Probl Diagn Radiol.

[10] Giess CS, Ip IK, Cochon LR, Gupte A, Dudley JC, Boland GW (2020). Predictors of self-reported burnout among radiology faculty at a large academic medical center. JACR.

[11] Fennessy FM, Mandell JC, Boland GW, Seltzer SE, Giess CS (2021). Strategies to increase resilience, team building, and productivity among radiologists during the COVID-19 era. JACR.

[12] Sood A, Sharma V, Schroeder DR, Gorman B (2014). Stress management and resiliency training (SMART) program among Department of Radiology faculty: a pilot randomized clinical trial. Explore.

[13] Gröschke D, Hofmann E, Müller ND, Wolf J. Individual and organizational resilience—insights from healthcare providers in Germany during the COVID-19 pandemic. Front Psychol. 2022;13. 10.3389/fpsyg.2022.965380 PMC9453859 36092080

[14] Liang F, Cao L (2021). Linking employee resilience with Organizational Resilience: the roles of coping mechanism and managerial resilience. Psychol Res Behav Manag.

[15] AMA. Creating a resilient organization for health care workers during a crisis 2020 [updated May 8th, 2020. Available from: https://www.ama-assn.org/practice-management/sustainability/creating-resilient-organization-health-care-workers-during.

[16] Campbell-Sills L, Stein MB. Psychometric analysis and refinement of the connor–davidson resilience scale (CD‐RISC): Validation of a 10‐item measure of resilience. JTS. 2007;20(6):1019-28. 10.1002/jts.20271 18157881

[17] Brown KW, Ryan RM (2003). The benefits of being present: mindfulness and its role in psychological well-being. J Pers Soc Psychol.

[18] Whitman R, Kachali Z, Roger H, Vargo D, Seville J (2013). Short-form version of the Benchmark Resilience Tool (BRT-53). MBE.

[19] Parikh JR, Bender CE (2021). How Radiology leaders can address Burnout. JACR.

[20] Anderson FA, Wheeler HB, Goldberg RJ, Hosmer DW, Forcier A, Patwardhan NA (1994). Changing clinical practice: prospective study of the impact of continuing medical education and quality assurance programs on use of prophylaxis for venous thromboembolism. Arch Intern Med.

[21] Bandong AN, Mackey M, Leaver A, Ingram R, Sterling M, Ritchie C (2019). An interactive website for whiplash management (my Whiplash Navigator): process evaluation of design and implementation. JMIR Formative Research.

[22] Cobo ME, Vicente A, Corres J, Royuela A, Zamora J (2009). Implementing a guideline for the request of chest and abdominal x-rays in nontrauma pathologic conditions in an ED. Am J Emerg Med.

[23] Headrick LA, Speroff T, Pelecanos HI, Cebul RD (1992). Efforts to improve compliance with the National Cholesterol Education Program guidelines: results of a randomized controlled trial. Arch Intern Med.

[24] Kovacs E, Strobl R, Phillips A, Stephan A-J, Müller M, Gensichen J (2018). Systematic review and meta-analysis of the effectiveness of implementation strategies for non-communicable Disease guidelines in primary health care. J Gen Intern Med.

[25] Gransjøen AM, Wiig S, Lysdahl KB, Hofmann BM (2018). Barriers and facilitators for guideline adherence in diagnostic imaging: an explorative study of GPs’ and radiologists’ perspectives. BMC Health Serv Res.

[26] Gransjøen AM, Wiig S, Lysdahl KB, Hofmann BM (2020). Health care personnel’s perception of guideline implementation for musculoskeletal imaging: a process evaluation. BMC Health Serv Res.

[27] Patriarca R, Di Gravio G, Costantino F, Falegnami A, Bilotta F (2018). An Analytic Framework to assess Organizational Resilience. Saf Health Work.

[28] Serrat O. Knowledge solutions: tools, methods, and approaches to drive organizational performance. Springer Nature; 2017.

[29] Chiappetta M, D’Egidio V, Sestili C, Cocchiara RA, La Torre G. Stress management interventions among healthcare workers using mindfulness: a systematic review. Senses & Sciences. 2018;5(2).

[30] Melo JACd, Gelbcke FL, Amadigi FR, Huhn A, Silva Cd, Ribeiro G. Psychological exhaustion of radiological nursing workers in nuclear medicine services. Rev Bras Enferm. 2021;73. 10.1590/0034-7167-2020-0169 33470380

